# Chemistry of Medicinal Plants, Foods, and Natural Products 2015

**DOI:** 10.1155/2015/121849

**Published:** 2015-10-18

**Authors:** Shixin Deng, Shao-Nong Chen, Jian Yang

**Affiliations:** ^1^Research and Development Department, Morinda Inc., American Fork, UT 84003, USA; ^2^UIC/NIH Center for Botanical Dietary Supplements Research, Department of Medicinal Chemistry and Pharmacognosy, College of Pharmacy, University of Illinois, Chicago, IL 60612, USA; ^3^Western Pacific Tropical Research Center, College of Natural and Applied Sciences, University of Guam, Mangilao, GU 96923, USA

Medicinal plants and natural products have been demonstrated to possess diversified health benefits for thousands of years by traditional uses and modern scientific research. An increased global demand has been observed over years. In 2014, we published the first special issue of Chemistry of Medicinal Plants, Foods, and Natural Products, aiming to address safety, identity, and efficacy of the natural entities. As a continuing program, we edited a special issue for 2015. The challenge for raw materials and finished products of botanicals attracts public and scientific communities. The safety and efficacy of these medicinal natural products are closely associated with their identity, authenticity, and quality, which in turn relate to many factors, such as geographical conditions (soil, sunlight, precipitation, and air) and postgrowth factors (harvesting, storage, transportation, manufacturing processes, etc.). In this special issue, we have invited original research articles addressing the novel analytical method development and validation, methodology and instrumentation improvement, chemical characterization, and biological activities of plant materials, extracts, and pure phytochemicals.

Sesquiterpene lactones represent a large group of natural compounds with diversified biological activities. B. Ivanescu et al. presented an overview of methodology on chemical extraction, identification, and quantification, as well as a literature review of biological activities of sesquiterpene lactones found in* Artemisia *genus. Z. Nagy et al. investigated variation of polyphenols, capsaicinoids, and vitamin C in six hybrids of chili peppers with HPLC during ripening period. By means of different detectors, 7 major capsaicinoids, many polyphenols, and vitamin C were identified and quantified. Based on these results, authors observed that the amounts of vitamin C increased and most of polyphenols kept the same level in all hybrids, while major capsaicinoids variation depended on different hybrid peppers during ripening. J. K. Kim et al. addressed the comparison of the nutritional and chemical properties and sensory attributes of* Seomae *mugwort and the commonly consumed species* Artemisia princeps* Pamp. and concluded that* Seomae *mugwort had higher contents of polyunsaturated fatty acids, total phenolic compounds, vitamin C, and essential amino acids and a better radical scavenging activity and more diverse volatile compounds than* A. princeps*.

Q. Wu and X. Gong used LabVIEW, a G language-based virtual instrument software, to establish a system for determination of sugar contents in honey. They stated that the new system may improve the accuracy of the measurement results by avoiding the artificial operation, cumbersome data processing, and the artificial error in optical activity measurement, and thus, it may apply to the analysis of the batch inspection on the sugar degree of honey. M. Dyduch-Siemińska et al. presented their research on the chemical composition of three wild strawberry cultivars fruits. They observed significant differences among these cultivars in terms of physicochemical property, flavonoids contents, phenolic acids, and total tannins and anthocyanins, as well as their antioxidant activity by means of the DPPH method. C. Corsaro et al. presented a method of using ^1^H HR-MAS NMR technique to investigate metabolites of Mediterranean diet. Authors processed and analyzed the HR-MAS solid-state NMR data with different software for PCA and quantitative analysis. By combination with qNMR techniques and PCA analysis, authors could distinguish the characterization of Protected Geographical Indication (PGI), Protected Designation of Origin (PDO), and Traditional Italian Food Products (PAT) vegetables from other non-PGI, non-PDO, and non-PAT. The advantage of HR-MAS NMR methodology is the rapidity and simultaneity of the qualitative and quantitative analyses without additional sample treatment. Although its sensitivity is not very high, the advantage of this methodology will make it very attractive for food industry.

G. Negri et al. discussed safety issue of homemade alcoholic beverages (unrecorded) in Brazil. By means of different measurements, including GC-FID/MS, FT-IR, and ICP-ASE, authors investigated a total of 152 samples collected from two cities of Sao Paulo State and seven cities of Minas Gerais State, Brazil. The results revealed that most of these unrecorded alcohol beverages have exceeded the regulatory limitation, including methanol and cyanates. Authors suggested more severe Quality Control (QC) and regulations on these unrecorded beverages are needed. S. Bado et al. investigated physical and chemical variability of tigernuts (*Cyperus esculentus*) cultivated in Burkina Faso. They reported that three* Cyperus esculentus* morphotypes studied are important source of macronutrients (starch, fat, and sucrose) and minerals (potassium, phosphorus, silicon, chlorine, sulfur, and magnesium). Genetic variability exists among cultivated tigernuts from Burkina Faso and from others grown worldwide. M. Godlewska et al. developed a precolumn derivatization method to determine the quantity of *α*-lipoic acid (LA), an organosulfur compound. In this method the LA degraded product, DHLA, was converted to 2-S-pyridinium derivative with 2-chloro-1-methylquinolinium tetrafluoroborate. It not only increases sensitivity with spectrophotometric assay, but also stabilizes the reaction product to increase reliable results for the determination of LA with HPLC method.

The paper authored by J.-H. Kim et al. developed and validated a quantitative analytical method by using high-performance liquid chromatography equipped with a photodiode array detector for determination of 19 marker compounds in herbal preparations. The method also was combined with a chemometric analysis involving principal component analysis and hierarchical clustering analysis. J. Chen et al. described that a Rapid-Resolution Liquid Chromatography- (RRLC-) Triple Quadrupole Mass Spectrometry with MRM has been developed for characterization of Deer-Horn Glue. It could be used for detection of gelatin adulteration quickly as QC of Deer-Horn Glue, a traditional Chinese medicine. M. M. Celik et al. addressed the protective effects of caffeic acid phenethyl ester (CAPE) and intralipid (IL) on nephrotoxicity caused by acute dichlorvos toxicity on rats. They concluded that both CAPE and IL may prevent the renal injuries with their antioxidant activities.

*Shixin Deng* *Shixin Deng* *Shao-Nong Chen* *Shao-Nong Chen* *Jian Yang* *Jian Yang*

